# Interferon-α2b Treatment for COVID-19 Is Associated with Improvements in Lung Abnormalities

**DOI:** 10.3390/v13010044

**Published:** 2020-12-30

**Authors:** Qiong Zhou, Michael R. MacArthur, Xinliang He, Xiaoshan Wei, Payam Zarin, Bola S. Hanna, Zi-Hao Wang, Xuan Xiang, Eleanor N. Fish

**Affiliations:** 1 Department of Respiratory and Critical Care Medicine, Union Hospital, Tongji Medical College, Huazhong University of Science and Technology, Wuhan 430022, China; zhouqiongtj@hust.edu.cn (Q.Z.); herbert1111@hust.edu.cn (X.H.); weixs@hust.edu.cn (X.W.); wz_hao_whuh@hust.edu.cn (Z.-H.W.); xxuan@hust.edu.cn (X.X.); 2 Department of Health Sciences & Technology, ETH Zurich, 8603 Zurich, Switzerland; mmacarthur@eth.ch; 3 Department of Immunology, Harvard Medical School, Boston, MA 02115, USA; payam_zarin@hms.harvard.edu (P.Z.); Bola_Hanna@hms.harvard.edu (B.S.H.); 4 Toronto General Hospital Research Institute, University Health Network and Department of Immunology, University of Toronto, Toronto, ON M5G 2M1, Canada

**Keywords:** COVID-19, interferon, CT images

## Abstract

Severe acute respiratory syndrome coronavirus 2 (SARS-CoV-2) infection causes coronavirus disease 2019 (COVID-19), a lung disease that may progress to systemic organ involvement and in some cases, death. The identification of the earliest predictors of progressive lung disease would allow for therapeutic intervention in those cases. In an earlier clinical study, individuals with moderate COVID-19 were treated with either arbidol (ARB) or inhaled interferon (IFN)-α2b +/−ARB. IFN treatment resulted in accelerated viral clearance from the upper airways and in a reduction in the circulating levels of the inflammatory biomarkers IL-6 and C-reactive protein (CRP). We have extended the analysis of this study cohort to determine whether IFN treatment had a direct effect on virus-induced lung abnormalities and also to ascertain whether any clinical or immune parameters are associated with worsening of lung abnormalities. Evidence is provided that IFN-α2b treatment limits the development of lung abnormalities associated with COVID-19, as assessed by CT images. Clinical predictors associated with worsening of lung abnormalities include low CD8+ T cell numbers, low levels of circulating albumin, high numbers of platelets, and higher levels of circulating interleukin (IL)-10, IL-6, and C-reactive protein (CRP). Notably, in this study cohort, IFN treatment resulted in a higher percentage of CD8+ T cells, lower tumor necrosis factor (TNF)-α levels and, as reported earlier, lower IL-6 levels. Independent of treatment, age and circulating levels of albumin and CRP emerged as the strongest predictors of the severity of lung abnormalities.

## 1. Introduction

In response to the global pandemic of coronavirus disease 2019 (COVID-19), there have been unprecedented efforts to develop therapeutic interventions, in the sphere of both vaccines and therapeutics. Presently, there are 200 vaccine candidates under preclinical evaluation, 23 in Phase 1 clinical trials, 14 in Phase 2, 7 in Phase 3, and 2 approved [1]. Different strategies have been employed to target the severe acute respiratory syndrome coronavirus 2 (SARS-CoV-2) virus, including direct inhibition of viral replication by targeting the RNA-dependent RNA polymerase, proteinase, and S protein, blocking the interactions with the angiotensin-converting enzyme 2 (ACE2) entry receptor and with the cellular serine protease, TMPRSS2, and regulating the host immune response to enhance viral clearance [2,3]. In the absence of a SARS-CoV-2 vaccine or its widespread availability, there is an urgent need for therapeutic interventions. Rather than developing a pathogen-specific intervention for each emerging and re-emerging global virus outbreak, with the potential for the development of drug resistance as the virus mutates, our strategy has been to focus on the host response to all viruses and consider the development of broad-spectrum antivirals. Type I interferons (IFNs), IFN-αs/ß, present as ideal candidates, because of their pleiotropic effects in directly targeting multiple stages of the virus replicative cycle and also activating an appropriate immune response to clear viruses, regardless of the type of virus infection [4].

In an earlier publication, we reported preliminary findings from a cohort of COVID-19 cases treated with inhaled interferon (IFN)-α2b [5]. Specifically, IFN-α2b treatment led to accelerated viral clearance from the respiratory tract and to a reduction in the serum levels of the inflammatory biomarkers interleukin (IL)-6 and C-reactive protein (CRP). The data suggested that by reducing viral shedding, IFN-α2b treatment may limit progression to severe disease. Indeed, there is accumulating evidence that the SARS-CoV-2 blunts an IFN response [6,7,8,9], and a poor IFN response is associated with severe and critical COVID-19 cases [10]. Thus, treatment with IFN may override the virus-mediated inhibition of the IFN response. Notably, to date there are more than 51 clinical trials of IFN treatment for COVID-19.

Chest computed tomography (CT) imaging abnormalities are associated with COVID-19 [11]. CT findings vary according to the time course of the infection, yet a pattern of ground-glass and consolidative pulmonary opacities, often with a bilateral and peripheral lung distribution, is emerging as the chest CT characteristic of COVID-19 infection. Accumulating data point to ground-glass abnormalities in early disease, with increasing consolidation later in the disease course [12,13,14,15]. Little is known of the biomarkers that correlate with CT imaging abnormalities and of whether IFN treatment may affect lung abnormalities. The objective of this study was to investigate whether the therapeutic benefit of IFN treatment in the COVID-19 cases we reported on previously is reflected in a reduction in CT chest abnormalities. Further, we interrogated the different hematological measurements to determine whether specific parameters were predictive of CT image lung abnormalities.

## 2. Materials and Methods

### 2.1. Study Population and Treatments

The study participants were laboratory-confirmed COVID-19 cases admitted to Union Hospital, Tongii Medical College, Wuhan, China, during the period of 16 January–20 February 2020. Cases reported symptoms that included fever, chills, cough, sore throat, headache, nasal discharge, myalgia, fatigue, shortness of breath, and/or diarrhea. Each patient was asked to identify their date of symptom onset. At the discretion of the attending physician, patients received antiviral treatment with either IFN-α2b (Tianjin Sinobioway Biology, 5 million (m) IU/mL), ARB (arbidol hydrochloride/Uniferovir; Jiangsu Simcere Pharm. Co., 100 mg dispersible tablets), or a combination of IFN-α2b plus ARB, in accordance with the current practice guidelines at the hospital at that time. For the treatment, 5 m IU IFN-α2b (1 mL) were added to 2 mL of sterile water and introduced as an aerosol by use of a nebulizer and a mask. IFN-α2b treatment was b.i.d, i.e., 10 m IU/day. ARB treatment was 200 mg (2 tablets) t.i.d, i.e., 600 mg/day. Additional COVID-19 confirmed cases from Wuhan Temporary Shelter Hospital (2–17 February 2020), who were transferred to Union Hospital and treated with only ARB, were included in the study. Ethics approval for the analysis of all data collected was waived by hospital Institutional Review Boards, since all patient data collected conformed with the policies for a public health outbreak investigation of emerging infectious diseases issued by the National Health Commission of the People’s Republic of China.

### 2.2. Laboratory Tests

Throat swab specimens were tested by real-time polymerase chain reaction (RT-PCR) for SARS-CoV-2, as described [5]. Samples were designated as positive (+) or negative (−) based on a threshold adjusted to fall within the PCR exponential phase, for both the open reading frame (ORF)1ab and N target genes. Complete blood count and serum biochemical tests were assessed as per the Union Hospital’s routine clinical laboratory procedures. Serum cytokine levels (IL-2, IL-4, IL-6, IL-10, tumor necrosis factor (TNF)-α, IFN-γ) and peripheral blood cell populations were measured throughout the course of the infection for all cases, as described [5].

### 2.3. CT Examination and Imaging Evaluation

All CT scans were conducted on patients in a supine position, using either a Philips Ingenuity Core128 (Philips Medical Systems, Best, The Netherlands) or a Somatom Definition AS (Siemens Healthineers, Forchheim, Germany), using a single inspiratory phase. To limit any motion artifacts, patients were instructed on breath holding, and chest CT images were then acquired during a single breath hold. For CT acquisition, the tube voltage was 120 kVp with automatic tube current modulation. From the raw data, CT images were reconstructed with a matrix size of 512 × 512 as axial images (thickness, 1.5 mm; increment, 1.5 mm) in transverse slice orientation with either hybrid iterative reconstruction (iDose level 4, Philips Medical Systems) or a pulmonary B70F kernel and a mediastinal B30f kernel (Siemens Healthineers). The mean CT dose index volume (+ standard deviation) was 8.4 mGy ± 2.0 (range, 5.2–12.6 mGy). Images were analyzed by two experienced physicians (HXL, WXS). All Digital Imaging and Communications in Medicine (DICOM) images from the CT studies were analyzed without access to clinical or laboratory findings. The evaluators independently and freely assessed the CT features using both axial CT images and multi-planar reconstruction images. After separate evaluations, any reconstructed images were transmitted to the workstation and picture archiving and communication systems (PACS) for multiplanar reconstruction post-processing. The semi-quantitative scoring system employed to evaluate pulmonary abnormalities was as described [15]. Briefly, each of the five lung lobes was visually scored on a scale of 0 to 5, with 0 indicating no involvement; 1, <5% involvement; 2, >5–25% involvement; 3, 26–49% involvement; 4, 50–75% involvement; and 5, >75% involvement. The total CT score was the sum of the individual lobar scores and ranged from 0 (no involvement) to 25 (maximum involvement). Representative CT images and corresponding scores are provided in Supplementary Figure S1.

### 2.4. Statistical Analyses

All analyses were performed using R version 4.0.2. Due to variability in time-point samples among patients, data for time course analyses were aligned to the date that treatment with IFN or ARB started. Due to the longer time period between CT scans and time to resolution of the lung pathology, time course data were binned into 10-day increments for analysis. Differences between IFN and ARB groups over time were compared using either the non-parametric Mann–Whitney U test with Holm’s correction for multiple comparisons or the Wilcoxon signed-rank test, as indicated.

For correlational and modeling analyses, data were binned into time intervals based on the CT scans. The beginning of the time interval was considered as the date of the first CT scan. The next time interval began on the date of the subsequent CT scan, and the final time interval ended on the date of hospital discharge. Non-CT data were averaged over the time-interval periods. Correlation coefficients were computed using non-parametric Spearman correlation coefficients.

## 3. Results

**Figure 1:**
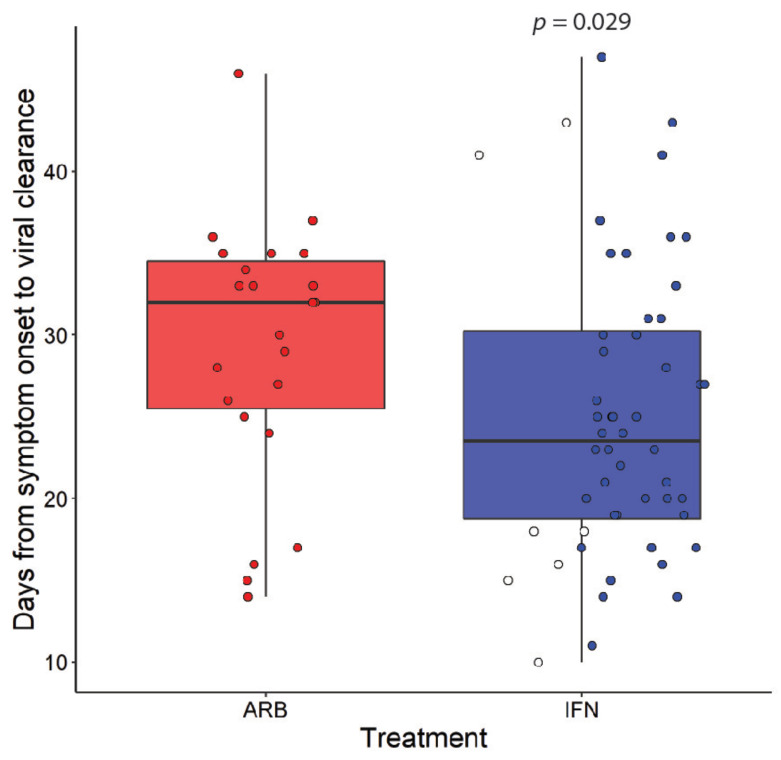
Interferon (IFN)-α2b treatment accelerates viral clearance. Coronavirus disease 2019 (COVID-19) cases were treated with either arbidol (ARB) alone (ARB, *n* = 24) or IFN-α2b with or without ARB (IFN, *n* = 53). Data points for cases treated with IFN alone are marked as open circles. Time to viral clearance from symptom onset is indicated; *p* value was calculated using Wilcoxon signed-rank test. These plots are re-derived from published data employing different population parameters [5].

In a recent exploratory clinical study conducted in Wuhan, China, at the start of the pandemic, we evaluated the therapeutic effects of inhaled IFN-α2 treatment in COVID-19 cases. In total, 77 adults hospitalized with confirmed COVID-19 were treated with nebulized IFN-α2b (*n* = 7), arbidol (ARB) (*n* = 24), or a combination of IFN-α2b plus ARB (*n* = 46). Serial COVID-19 testing and CT scans, along with hematological measurements, including cell counts, blood biochemistry and serum cytokine levels, and temperature and blood oxygen saturation levels, were recorded for each patient during their hospital stay. Treatment with IFN-α2b significantly reduced the levels of detectable virus in the upper respiratory tract, on average by 7 days (Figure 1) and, in parallel, reduced the elevated blood levels of the inflammatory biomarkers IL-6 and CRP [5].

### 3.1. Lower CT Scores in COVID19 Patients Following IFNa2b Treatment

We extend these preliminary analyses to consider the effects of IFN-α2b treatment on CT findings of lung abnormalities associated with COVID-19. We compared the CT scores of those patients who received only ARB (*n* = 24) with those of patients who received either IFN-α2b alone (*n* = 7) or IFN-α2b + ARB (*n* = 46). CT scans were performed using a single inspiratory phase and retrospectively scored by two experienced physicians who were at the time blind to clinical and laboratory findings.

**Figure 2:**
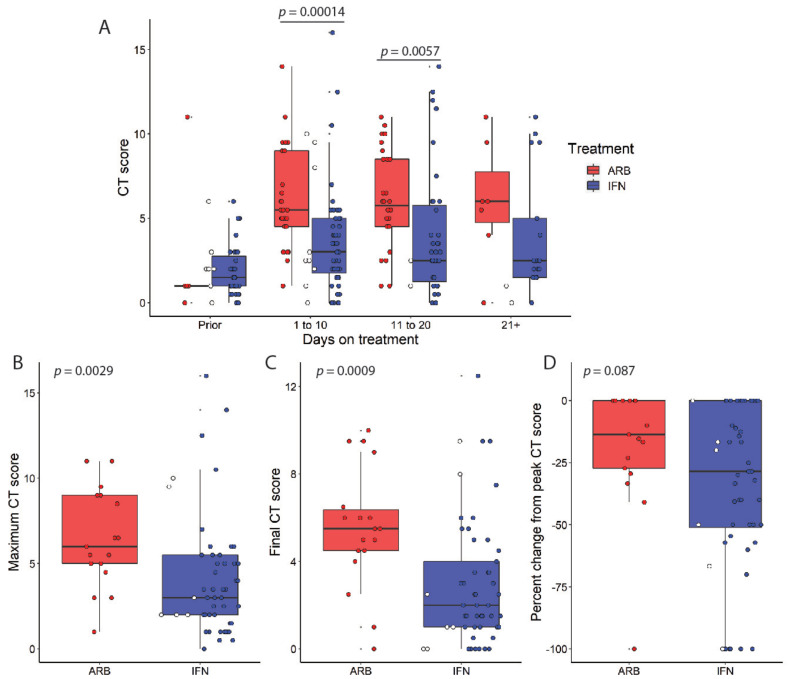
IFN-α2b treatment reduces the severity of lung abnormalities in CT scans during the course of COVID-19. (**A**) COVID-19 cases were treated with either ARB alone (ARB, *n* = 24) or IFN-α2b with or without ARB (IFN, *n* = 53). Data points for those cases treated with IFN alone are marked as open circles. Chest CT scores were recorded relative to treatment onset, as indicated. Each data point represents a score from a CT image; *p* values were calculated using thenon-parametric Mann–Whitney U test with Holm’s correction for multiple comparisons. Maximum (**B**), final (**C**), and (**D**) % change from peak CT scores are shown with *p* values calculated using Wilcoxon signed-rank test.

IFN- or ARB-treated patient CT scores were batched based on the elapsed time from treatment (Figure 2A). The timing of CT scans relative to the start of treatment was not consistent for all patients, as the attending clinician requested CT scans for each patient when the patient’s clinical status suggested worsening or improvements in the respiratory status. Refer to Supplementary Figure S2 for time-course plots of CT scans for all patients. Despite there being a limited number of CT scans immediately prior to the onset of treatment, most notably in those patients who went on to receive ARB alone, all COVID-19 patients exhibited comparable CT scores, on average <2.0. Indeed, if CT images on day 1 of treatment were included for baseline consideration, again there were no statistically significant differences in the CT scores (Supplementary Figure S3). On day 1 of treatment, one would not anticipate a treatment effect on lung abnormalities. Refer to Supplementary Figure S1 for representative CT images and scores. The CT scores were significantly different over the disease course for patients who received IFN compared with those of patients who did not. Specifically, average CT scores remained low in IFN-α2b-treated patients compared with those of ARB-treated patients, whose average CT scores were consistently higher, up to at least 20 days following treatment. In addition, IFN-α2b-treated patients had significantly lower peak CT scores compared to ARB-treated COVID-19 patients (Figure 2B), and their final CT scores prior to discharge were significantly lower for IFN-treated patients in comparison to the ARB-treated group (Figure 2C). This finding was further supported by a trend of greater reduction from the peak CT score observed for IFN-treated patients versus those treated with ARB (Figure 2D).

### 3.2. CT Score Severity Correlates with Clinically Relevant Biomarkers of Disease Severity

**Figure 3:**
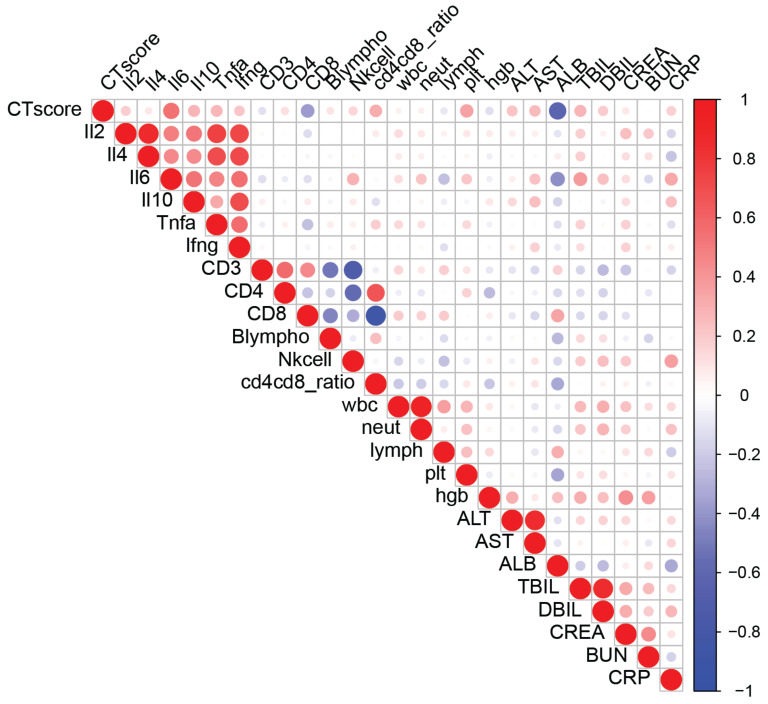
Lung CT image abnormalities correlate with many clinically relevant biomarkers of COVID-19 severity. Spearman’s correlation coefficients were calculated between blood-based biomarkers measured and CT scores. Blue-to-red heat map and dot sizes identify levels of correlation. Interleukin (IL)2, IL-2; IL4, IL-4; IL6, IL-6; IL10, IL-10; Tnfa, TNF-α; Ifng, IFN-γ; Blympho, B lymphocytes; Nkcell, NK cells; wbc, white blood cells; neut, neutrophils; lymph, lymphocytes; plt, platelets; hgb, hemoglobin; ALT, alanine transaminase; AST, aspartate aminotransferase; ALB, albumin; TBIL, total-value bilirubin; DBIL, direct bilirubin; CREA, creatinine; BUN, blood urea nitrogen; CRP, C-reactive protein.

Cognizant that the sensitivity of chest CT scans provides an accurate determination of COVID-19 disease severity and is useful to decide patient management [16,17,18,19], we examined whether the CT scores correlated with clinical and laboratory biomarkers in our patient cohort, independent of treatment (Figure 3), Table 1.

**Figure 4:**
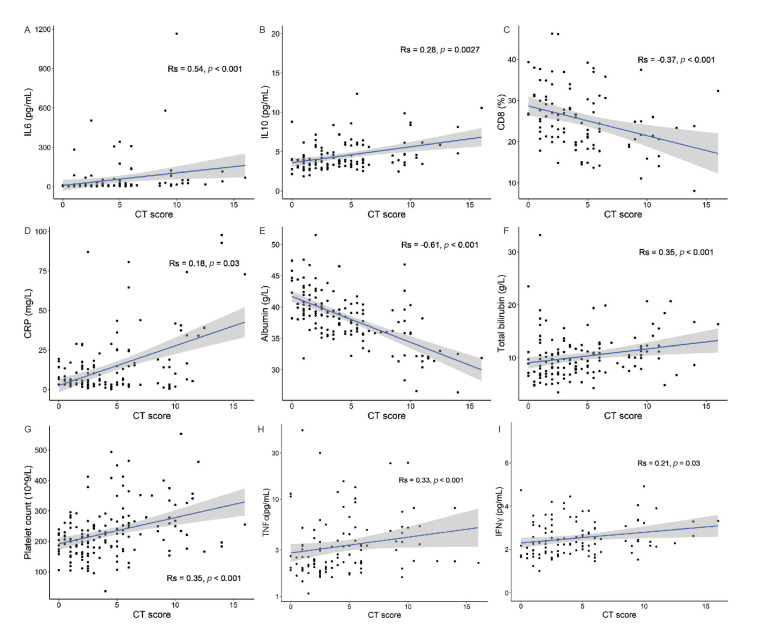
The severity of lung abnormalities is associated with specific immune and clinical biomarkers. Scatterplots depicting associations between CT scores and various blood-based biomarkers. Spearman’s correlation coefficients were calculated between CT scores and (**A**) IL-6, (**B**) IL-10, (**C**) CD8+ T cells, (**D**) CRP, (**E**) albumin, (**F**) total bilirubin, (**G**) platelets, (**H**) TNF-α, and (**I**) IFN-γ, as indicated. Shaded areas indicate 95% confidence intervals.

Employing Spearman’s correlation coefficient for inflammatory and clinical markers and immune cell populations revealed a distinct set of parameters that correlated with patient CT scores. Patient CT scores correlated with the levels of inflammatory cytokines including IL-6, TNF-α, IFN-γ, and IL-10 (Figure 3 and Figure 4A,H,I,B, respectively). In addition, the CT scores correlated with the percentage of CD8+ T cells calculated from total CD3+ lymphocytes, with a low percentage of CD8+ T cells corresponding to high CT scores (Figure 4C). In agreement with reports of platelet dysregulation and increased coagulation in COVID-19 patients [20], the chest CT scores had a strong and significant correlation (R_S_ = 0.35, *p* <0.001) with platelet counts in our cohort (Figure 4G).

Elevated levels of CRP are a common feature of unresolved inflammation following respiratory infections [21,22,23]. We provide evidence of a direct correlation between CRP levels and chest CT scores (Figure 4D). Of particular interest, serum albumin was recorded as the biomarker with the strongest correlation with CT scores in our cohort, with a majority of patients with a CT score higher than 10 displaying a sharp drop in serum albumin levels below the normal range of 34–54 g/L (Figure 4E). In agreement with a drop in albumin levels, higher CT scores correlated with increases in serum bilirubin levels (Figure 4F). The data in Supplementary Figure S4 illustrate that the correlations of the CT scores with the levels of inflammatory and clinical markers and immune cell populations persist when the data are segregated for ARB- and IFN-treated cases.

### 3.3. Mixed-Effects Modeling Reveals CRP, Albumin, and Age as the Strongest Predictors of CT Scores

To identify an unbiased group of parameters that can collectively predict CT scores, we generated a linear mixed-effects model with CT score as the outcome variable, patient ID as a random effect, and parameters that were shown to significantly correlate with CT scores as fixed effects (IL-6, IL-10, CRP, albumin, and platelet count). Age and sex were also included as fixed effects. During model assessment, other biomarkers with high co-linearity (those with a variance inflation factor greater than 3) were removed from the model. Using this model, age had a significant positive predictive value (0.059 ± 0.019, *p* = 0.003), and so did CRP (0.041 ± 0.014, *p* = 0.008). Albumin had a significant negative predictive value (−0.16 ± 0.07, *p* = 0.028). Other parameters did not reach statistical significance and showed large relative error estimates. To estimate the *p*-values, we used a t-test with Satterthwaite approximations of degrees of freedom.

### 3.4. IFN Effects on Critical Predictors of Worsening Lung Abnormalities

**Figure 5:**
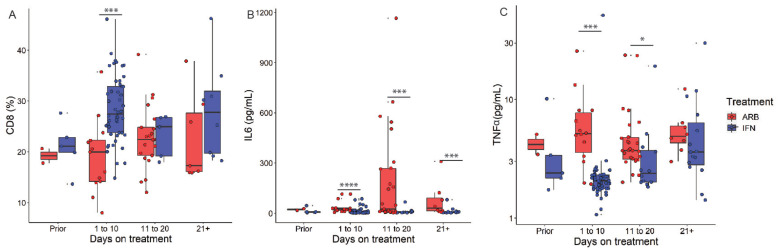
IFN-α2b treatment regulates the levels of clinical and immune biomarkers associated with lung CT image abnormalities. COVID-19 cases were treated with either ARB alone (ARB, *n* = 24) or IFN-α2b with or without ARB (IFN, *n* = 53). % CD8+ T cells (**A**) and levels of IL-6 (**B**) and TNF-α (**C**) are depicted across the time course of treatment with either ARB or IFN; *p* values were calculated using the non-parametric Mann–Whitney U test with Holm’s correction for multiple comparisons. * *p* < 0.05, *** *p* < 0.001, **** *p* < 0.0001.

We next examined the effects of IFN treatment on the identified predictors of worsening lung abnormalities. We considered only those biochemical or immune cell populations whose levels prior to the onset of treatment were the same in all patients. The data in Figure 5 revealed that IFN treatment prevented virus-induced reductions in CD8+ T cells and confirmed our earlier findings of IFN treatment reducing the levels of IL-6 [5]. We also provide evidence that IFN treatment limited virus-induced increases in TNF-α levels.

The examination of circulating CRP levels in IFN-treated and ARB-treated patients prior to the start of treatment revealed that those patients who were administered IFN (+/−ARB) had significantly higher CRP levels prior to treatment onset than those who received only ARB treatment. Although the cumulative data show that IFN treatment had a greater effect on reducing CRP levels than ARB alone [5], herein CRP data analysis is excluded, given the inclusion criterion of similar levels in all patients prior to treatment onset.

## 4. Discussion

The COVID-19 pandemic has highlighted the impact of this disease on hospital capacity, specifically on intensive care unit and supplemental oxygen/ventilator capacities. There is an urgent need of clinical predictors of prognosis to permit early interventions with approved therapeutics. Increasing evidence identifies chest CT imaging as a good correlate for disease severity and short-term prognosis in COVID-19 patients. CT scans provide a quantitative measurement of lung pathology, in contrast to measurements of circulating levels of various analytes and cytokines, that may identify inflammation, though not specifically in the target organ for this respiratory virus infection.

In this exploratory study, IFN-α2 treatment led to significantly lower CT scores, compared to treatment with ARB alone. This finding supports our earlier evidence of accelerated viral clearance from the upper respiratory airways for this cohort of COVID-19 patients treated with inhaled IFN-α2b [5]. We next examined whether specific clinical measurements were associated/correlated with CT scores, independent of treatment, to determine whether there are circulating factors directly linked to lung abnormalities in COVID-19.

We provide evidence that a higher percentage of CD8+ T cells correlated with lower CT scores, in agreement with a role for CD8+ lymphocytes in viral clearance [2]. Consistent with emerging data that serum IL-10 levels predict disease severity [24], higher levels of IL-10 correlated with higher CT scores in our study cohort. Among standard clinical biomarkers, higher serum platelet, bilirubin, and CRP levels all acted as strong predictors of a higher CT score, whereas low serum albumin correlated with higher CT score severity. Abnormal platelet numbers suggest coagulation abnormalities, and atypical albumin and bilirubin levels reflect organ dysfunction. Low serum albumin is associated with severe COVID-19 [25]. Using a mixed-effects model, which held CT scores as the fixed variable, we found that age and the levels of CRP and albumin were the strongest predictors of patient CT scores. Certainly, elevated CRP levels have consistently been linked to disease severity [26].

Lung abnormalities are the hallmark of disease severity in COVID-19 patients. CT imaging provides a measurable quantitation of lung abnormalities. Viewed altogether, the findings from this exploratory clinical study suggest that early monitoring of two key predictors of lung abnormalities, namely, circulating levels of CRP and albumin, may permit early therapeutic interventions to avoid or limit COVID-19 progression. Moreover, our data indicate that IFN treatment protects against worsening lung abnormalities in COVID-19 patients and limits the effects of SARS-CoV-2 infection on reducing CD8+ T cells and increasing IL-6 and TNF-α levels.

## Figures and Tables

**Table 1:** Correlations between CT scores and blood biomarkers.

	All		ARB		IFN	
	Rs	*p*	Rs	*p*	Rs	*p*
IL-2	0.190	0.047	−0.017	0.919	0.047	0.696
IL-4	0.097	0.313	−0.343	0.032	−0.004	0.977
IL-6	0.542	0.000	0.110	0.506	0.434	0.000
IL-10	0.283	0.003	0.163	0.321	0.361	0.002
TNF-α	0.272	0.004	0.156	0.342	−0.030	0.801
IFN-γ	0.212	0.026	−0.092	0.579	0.172	0.151
CD3 cells	−0.114	0.265	0.300	0.076	−0.265	0.037
CD4+ T cells	0.119	0.243	0.312	0.064	0.038	0.768
CD8+ T cells	−0.371	0.000	−0.292	0.085	−0.349	0.005
B lymphocytes	0.106	0.338	0.046	0.844	0.158	0.219
NK cell	0.159	0.152	−0.267	0.242	0.166	0.198
CD4:CD8 ratio	0.313	0.002	0.302	0.074	0.252	0.049
white blood cells	0.058	0.478	−0.019	0.909	−0.098	0.301
neutrophils	0.066	0.419	0.031	0.853	−0.099	0.297
lymphocytes	−0.093	0.256	−0.088	0.601	−0.113	0.230
platelets	0.355	0.000	0.200	0.229	0.303	0.001
hemoglobin	−0.125	0.124	0.190	0.254	−0.183	0.051
alanine transaminase	0.229	0.006	0.406	0.013	0.268	0.006
aspartate aminotransferase	0.255	0.002	0.541	0.001	0.251	0.010
albumin	−0.620	0.000	−0.292	0.079	−0.701	0.000
total bilirubin	0.286	0.001	−0.037	0.829	0.215	0.028
direct bilirubin	0.197	0.019	−0.110	0.518	0.201	0.040
creatinine	0.093	0.270	−0.078	0.647	−0.027	0.781
blood urea nitrogen	0.007	0.931	−0.064	0.706	−0.037	0.708
glucose	0.499	0.000	0.316	0.684	0.459	0.000
CRP	0.179	0.033	0.290	0.073	0.235	0.017

Table showing Spearman correlation coefficients (Rs) and *p* values between CT score and various blood-based markers in the entire cohort (All) or in the ARB or IFN treatment groups, separately. Color scale indicates the correlation coefficient value and corresponds to the heat map in Figure 3**.**

## Data Availability

The raw data supporting this study will be made available by the corresponding author without undue reservation, to any qualified researcher.

## Supplementary Materials

The following are available online at https://www.mdpi.com/1999-4915/13/1/44/s1, Figure S1: CT examination and imaging evaluation, Figure S2: Time-point plots of CT scores of CT scans for individual patients, Figure S3: IFN-α2b treatment reduces the severity of CT lung abnormalities during the course of COVID-19, Figure S4: Severity of lung abnormalities correlates with specific immune and clinical biomarkers.
