# Supplementary material for: Interferon-α2b Treatment for COVID-19 Is Associated with Improvements in Lung Abnormalities
Source: Viruses. 2020 Dec 30;13(1):44. doi: 10.3390/v13010044 (PMC7824252; doi:10.3390/v13010044)
Supplement: Supplementary file 1 [file viruses-13-00044-s001.pdf]

Supplementary Figure S1

CT examination and imaging evaluation

All CT scans were conducted in patients in a supine position, using either a Philips Ingenuity Core128 (Philips Medical Systems, Best, The Netherlands) or a Somatom Definition AS (Siemens Healthineers, Erlangen, Germany), using a single inspiratory phase. To limit any motion artifact, patients were instructed on breath holding, and chest CT images were then acquired during a single breath hold. For CT acquisition, the tube voltage was 120 kVp with automatic tube current modulation. From the raw data, CT images were reconstructed with a matrix size of 512 × 512 as axial images (thickness, 1.5 mm; increment, 1.5 mm) in transverse slice orientation with either hybrid iterative reconstruction (iDose level 5, Philips Medical Systems) or a pulmonary B70F kernel and a mediastinal B30f kernel (Siemens Healthineers). The mean CT dose index volume (± standard deviation) was 8.4 mGy ± 2.0 (range, 5.2–12.6 mGy). Images were analyzed by two experienced physicians (HXL, WXS). All Digital Imaging and Communications in Medicine (DICOM) images from the CT studies were analyzed without access to clinical or laboratory findings. The evaluators independently and freely assessed the CT features using both axial CT images and multi-planar reconstruction images. After separate evaluations, any reconstructed images were transmitted to the workstation and picture archiving and communication systems (PACS) for multiplanar reconstruction post-processing. The semi-quantitative scoring system employed to evaluate pulmonary abnormalities was as described [15]. Briefly, each of the five lung lobes was visually scored on a scale of 0 to 5: 0 indicating no involvement; 1, <5% involvement; 2, >5%–25% involvement; 3, 26%–49% involvement; 4, 50%–75% involvement; and 5, >75% involvement. The total CT score was the sum of the individual lobar scores and ranged from 0 (no involvement) to 25 (maximum involvement). Representative CT images are provided. Individual patients are identified as P1–4. P1 (score 7) ground-glass opacity and consolidation in the subpleural regions of both lungs; P2 (score 11) ground-glass opacity with mixed consolidation in the subpleural regions of the left lower lobe; P3 (score 13) coexisting ground-glass opacity, patchy-like lesions, and consolidation as well as peribronchial and subpleural distribution; P4 (score 16) bilateral and peripheral predominant consolidation. Arrows indicate abnormalities identified for P1–4.

Appendix Figure 1

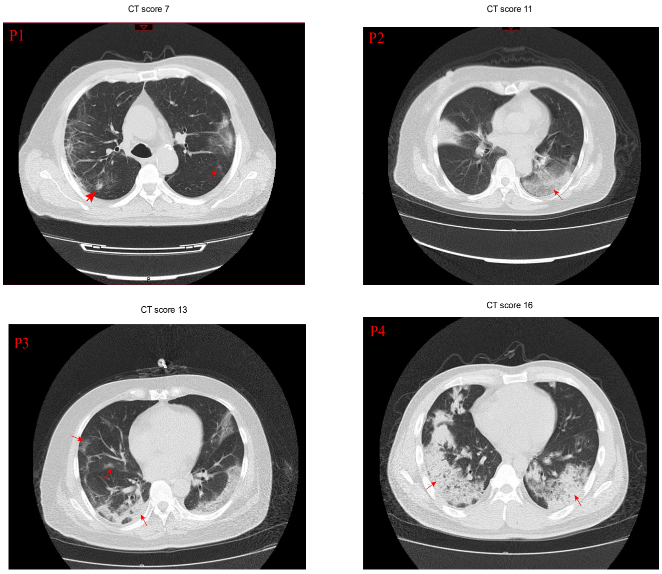

Supplementary Figure S2

Time-point plots of CT scores of CT scans for individual patients.

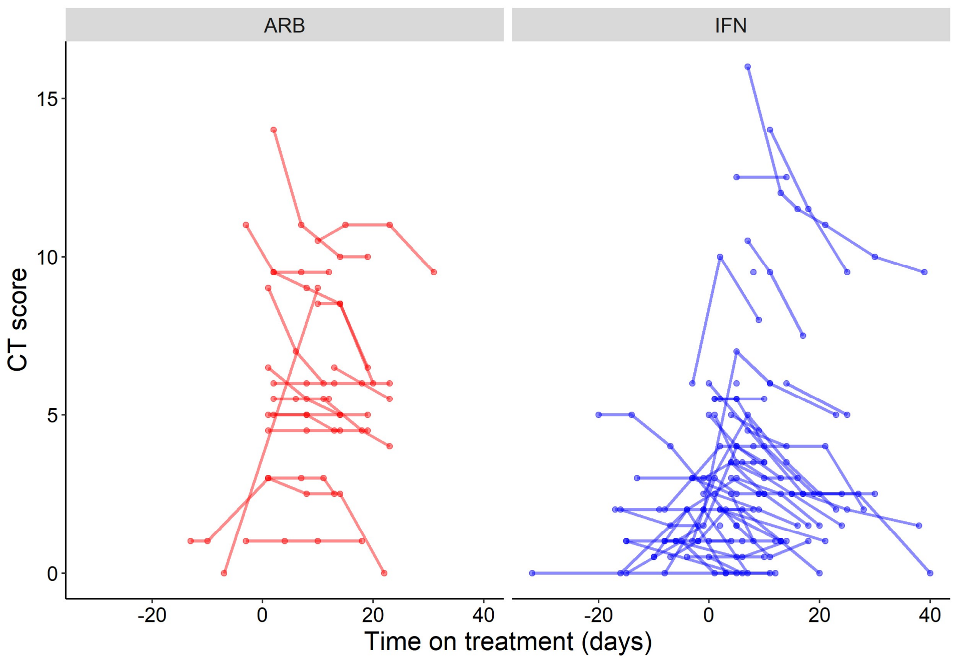

Each dot represents the time point and corresponding CT score. Single dots identify patients for which only one CT scan was taken.

Supplementary Figure S3

IFN-α2b treatment reduces the severity of CT lung abnormalities during the course of COVID-19. COVID-19 cases were treated with either ARB alone (ARB, n = 24) or IFN-α2b with or without ARB (IFN, n = 53). Chest CT scores were recorded relative to treatment onset, as indicated. Each data point represents a score from a CT image; p values were calculated using the non-parametric Mann–Whitney U test with Holm's correct multiple comparisons.

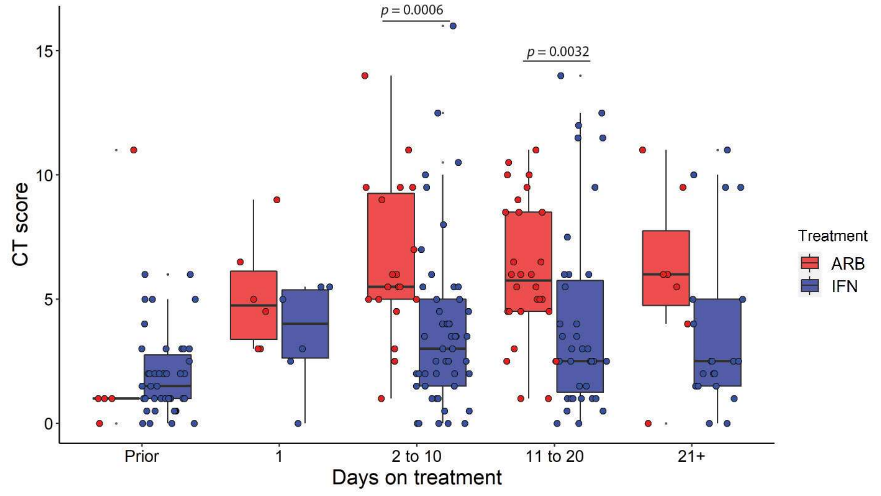

Supplementary Figure S4

Severity of lung abnormalities correlates with specific immune and clinical biomarkers. Scatterplots depicting associations between CT scores and various blood-based biomarkers for ARB- and IFN-treated (+/−ARB) patients. Spearman's correlation coefficients were calculated between CT scores and the blood analytes, cytokines, and immune cells indicated. Shaded areas indicate 95% confidence intervals of the respective ARB (red) and IFN (blue) plots.

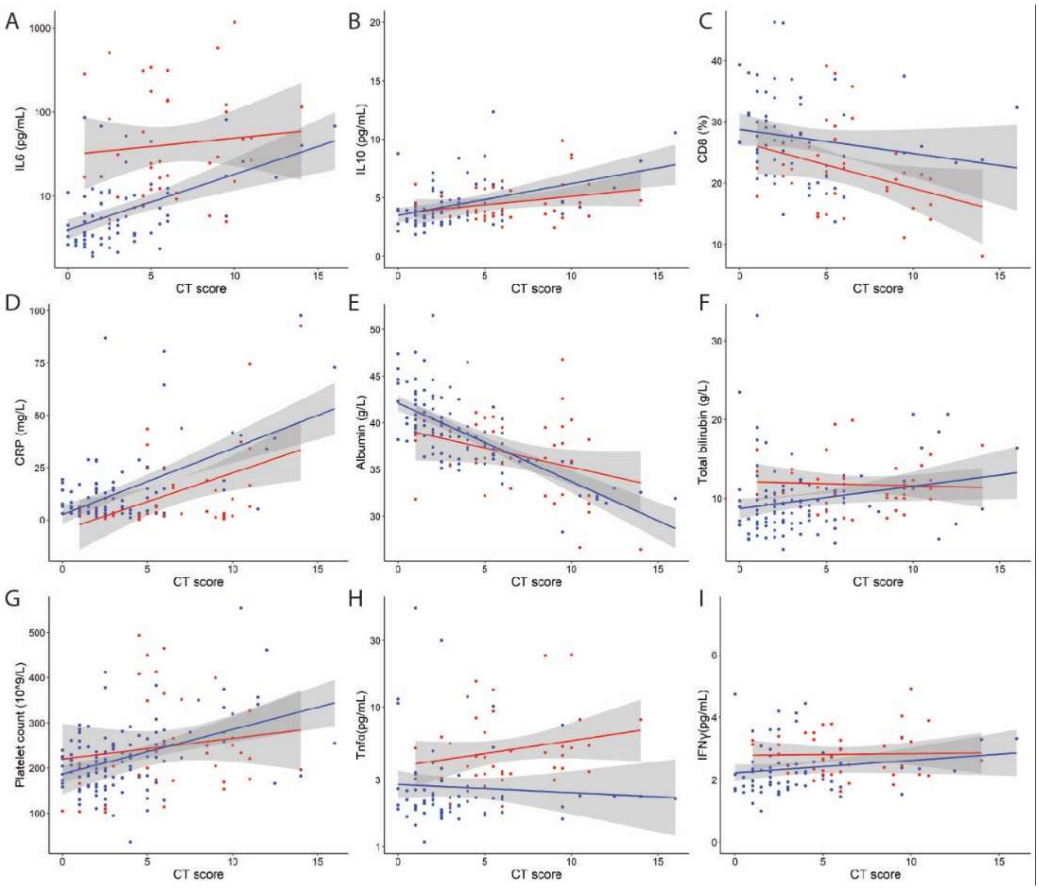
A
IL6 (pg/mL)
CT score
B
IL10 (pg/mL)
CT score
C
CD8 (%)
CT score
D
CRP (mg/L)
CT score
E
Albumin (g/L)
CT score
F
Total bilirubin (g/L)
CT score
G
Platelet count (10^9/L)
CT score
H
Tnfα(pg/mL)
CT score
I
IFNγ(pg/mL)
CT score
